# Low-dose methotrexate in sickle-cell disease: a pilot study with rationale borrowed from rheumatoid arthritis

**DOI:** 10.1186/s40164-017-0078-1

**Published:** 2017-06-19

**Authors:** Silvia R. Brandalise, Rosemary Assis, Angelo B. A. Laranjeira, José Andrés Yunes, Pedro O. de Campos-Lima

**Affiliations:** 1 Boldrini Children’s Center, Rua Dr. Gabriel Porto 1270, Cidade Universitaria, Campinas, SP 13083-210 Brazil; 2 Department of Pediatrics, School of Medicine, State University of Campinas, Campinas, SP Brazil; 3 Department of Psychology, Paulista University, Campinas, SP Brazil; 4 National Cancer Institute, National Institutes of Health, Bethesda, MD USA

**Keywords:** Sickle cell disease, Methotrexate, Inflammation, Pain, Chemokines

## Abstract

**Background:**

Inflammation is a major feature of sickle cell disease (SCD). Low-dose methotrexate (MTX) has long been used in chronic inflammatory diseases. This pilot study examined the MTX effect on acute vaso-occlusive pain crises (VOC) in SCD patients.

**Methods:**

Fourteen adults on hydroxyurea with severe and refractory VOC received one intramuscular injection of 10 mg of MTX per week for 12 weeks. A single weekly dose of 5 mg of leucovorin was administered orally 48 h after each MTX injection. The primary outcome was reduction in number/intensity of acute pain episodes. The secondary outcomes were improvement of quality of life (QOL) and reduction of the inflammatory status.

**Results:**

MTX did not significantly change the median VOC frequency (12 before vs 10.5 during treatment, *P* = 0.6240) or the median McGill pain index (45 at week 0 vs 39.5 at week 12, *P* = 0.9311). However, there was a decrease of ≥50% in chronic pain resulting from avascular osteonecrosis (AVN) in 5 out of 7 patients with radiologic evidence of AVN, with the perception of longer pain-free periods. There was a 44.4% median gain in physical function in the SF-36 QOL questionnaire (*P* = 0.0198). MTX treatment up-regulated two C-X-C motif chemokines (CXCL), CXCL10 (*P* = 0.0463) and CXCL12 (*P* < 0.0001), without significant effect on 14 additional plasma inflammatory markers. Adverse events: One individual had fever of unknown origin. Respiratory tract infections were recorded in five patients. Among the latter, one also had dengue fever and another had a central venous line infection and died of pneumonia and septic shock. Three patients with previous history of hydroxyurea-induced hematological toxicity developed low blood platelet counts while receiving simultaneously MTX and hydroxyurea.

**Conclusions:**

Although MTX did not reduce acute VOC frequency/intensity, it decreased chronic pain and led to QOL improvement.

*Trial registration* http://www.who.int/ictrp/en/ and http://www.ensaiosclinicos.gov.br, RBR-2s9xvn, 19 December 2016, retrospectively registered

**Electronic supplementary material:**

The online version of this article (doi:10.1186/s40164-017-0078-1) contains supplementary material, which is available to authorized users.

## Background

Vaso-occlusive crises (VOC) are important features of sickle cell disease (SCD), which is characterized by ischemic injury of potentially all major organs in the body [1–3]. Tissue damage initially results from hypoxia and then from oxygen re-exposure, that causes massive production of reactive oxygen species (ROS), local activation of endothelial cells, platelets, neutrophils, monocytes, resident tissue macrophages and perivascular mast cells [4]. Pro-inflammatory mediators derived from activated cells create a positive feedback that amplifies the vascular/tissue damage and inflammation [3, 4]. In about 50% of the adult patients, ischemic lesions evolve to avascular osteonecrosis often located in the femoral and humeral heads [5, 6].

Another important aspect of SCD is the chronic hemolysis occurrence [3, 7]. Intra-erythrocyte ADP and ATP released in the extracellular space contribute to vaso-occlusion and inflammation [7–9]. Similarly, free hemoglobin is a powerful NO scavenger and promotes ROS accumulation [3, 10]. In addition, the presence of the prosthetic heme group in plasma activates the coagulation system and innate immunity [11]. Heme acts as a ligand of the Toll-like receptor 4 (TLR4), with activation of two main pathways in endothelial cells [12]. The first one is the protein kinase C-mediated mobilization of Weibel–Palade bodies (WPBs) to the cell surface, simultaneously releasing the pro-coagulant von Willebrand Factor and loading P-selectin onto the cell membrane to promote leukocyte-endothelial binding and stasis [11]. The second pathway is MyD88-mediated NF-κB activation and subsequent transcription of several responsive genes, notably those encoding pro-inflammatory mediators, such as: IL-1, IL-6, IL-8, VCAM-1, ICAM-1, P-selectin and E-selectin [4, 11–13].

For decades, nonsteroidal anti-inflammatory drugs (NSAID) have been recommended for treatment of light and moderate pain in SCD patients [1, 14]. Similar to NSAID, low-dose methotrexate (MTX) exhibits anti-inflammatory activity that has been successfully used for therapy of several chronic inflammatory diseases, such as rheumatoid arthritis [15], psoriasis [16], uveitis [17], juvenile dermatomyositis [18], localized scleroderma [18], Crohn’s disease [18], Wegener granulomatosis [19], and sarcoidosis [20]. This study tests the hypothesis that the inflammatory component of sickle cell disease can be responsive to methotrexate treatment, with clinical improvement in frequency and pain intensity of VOC episodes.

## Methods

### Patients and study design

This was a prospective pilot study that enrolled young adult patients with sickle cell disease who were under chronic hydroxyurea treatment and had more than 3 severe VOC episodes/year, that were refractory to opioids for periods longer than 3 weeks duration. For inclusion purposes, a VOC episode was defined as any severe acute pain episode demanding emergency admission for opioid parenteral administration. Pregnancy and concomitant infection were exclusion criteria. Contraceptive measures were routinely advised for fertile female patients under chronic hydroxyurea. The study was conducted in the city of Campinas in the Brazilian Southeast region. The population in this metropolitan area is prevalently white but includes close to 25% individuals self-described as multiracial or black. About 230 SCD patients ≥18 years of age attend Boldrini Children’s Center each year. Recruitment for this study was opened for 10 months, during which 14 patients had severe disease and met the inclusion criteria. None of them declined to participate. Eleven carried HbSS and 3 had HbSC. There were 9 men and 5 women, with a median age of 23.5 years (range 18–32 years). Clinical and demographic data are presented in Table 1. All patients had experienced many more acute and severe pain crisis episodes than the inclusion threshold of 3/year, despite hydroxyurea treatment. One patient (#6) had 19 VOC episodes per month. The median number of VOC episodes in the other 13 patients was 3.3/month (95% CI 2.0–5.0). Seven patients presented avascular osteonecrosis of the femoral and/or humeral heads.

**Table 1:** Clinical and demographic data from the study patients

Patient	Age (years)	Gender	Genotype	Avascular necrosis	Previous VOC/month^b^
1	20	M	HbSS	Bilateral femoral heads	8
2	22	F	HbSC	No	1.3
3	25	F	HbSS	Right femoral head	4
4	20	F	HbSS	Bilateral femoral and humeral heads	1.3
5	20	F	HbSS	No	3
6^a^	32	M	HbSS	Bilateral femoral heads, right humeral head	19.3
7	21	M	HbSS	Right femoral head	2
8^a^	32	M	HbSS	Bilateral femoral and humeral heads	3.3
9	23	M	HbSC	Left femoral head	2.6
10	25	M	HbSS	No	7
11	18	M	HbSS	No	3.6
12	24	M	HbSS	No	3.3
13	24	F	HbSC	No	4.3
14	28	M	HbSS	No	5

^a^Patients with hip joint replacement

^b^VOC frequency was calculated from the trimester preceding the study recruitment

The experimental treatment consisted of one intramuscular injection of 10 mg of methotrexate per week for 12 weeks. A single weekly dose of 5 mg of leucovorin was administered orally 48 h after each methotrexate injection. Sickle cell disease is a condition characterized by hemolysis and increased erythropoiesis, which may compromise folate levels [7]. The rationale for leucovorin rescue in our protocol stems from the anti-folate activity of methotrexate. Leucovorin is a reduced form of folate that limits the potential toxic effects associated with the inhibition of folate-dependent enzymes by methotrexate. Interruption of ongoing treatment with hydroxyurea and opioids was not recommended. Hematological and biochemical parameters were routinely recorded.

The primary endpoint was the decrease in frequency and intensity of severe acute pain episodes that required emergency medical assistance during the 12-week long study, compared to an equivalent period preceding the MTX treatment.

The secondary endpoints were the blood levels of inflammatory markers and the quality of life (QOL), which were evaluated at three time points (0, 6 and 12 weeks) of MTX treatment. Each patient was considered as his/her own control. S.R.B, P.O.C.L., R.A., J.A.Y., and A.B.A.L. analyzed and had access to primary clinical data. This study followed the Declaration of Helsinki, was approved by the Institutional Ethics Committee, and was registered in the Brazilian National Ethics Database/Plataforma Brasil (CAAE: 36641414.9.0000.5376). All participants gave written informed consent. This study was registered at http://www.who.int/ictrp/en/ and http://www.ensaiosclinicos.gov.br (World Health Organization International Clinical Trials Registry Platform/Brazilian Clinical Trials Registry Identifier: RBR-2s9xvn).

### Pain measurement and QOL

The general effectiveness of methotrexate treatment was evaluated by the patients in a scale of 0–10 at the last follow-up visit. Pain quantification was performed with the Brazilian validation of the McGill Pain Questionnaire, which contains 68 pain descriptors [21, 22]. Considering that avascular osteonecrosis pain has a different course from acute painful crises, patients with that complication were asked to quantify separately the treatment efficiency on the associated chronic pain from 0 to 10. Chronic pain intensity was plotted on a scale, in which 0 represents no pain (Treatment efficiency: 10) and 1.0 represents the maximal baseline chronic pain (Treatment efficiency: 0).

Quality of life was assessed according to the Brazilian Portuguese validation of the SF-36 Questionnaire, which contains four 0–100 subscales for the physical and mental domains [23, 24]. Symptoms of depression were measured with the 21-item Beck inventory [25, 26].

### Laboratory methods for inflammatory markers

Blood samples (20 ml) were collected in EDTA tubes before the first meal. Plasma aliquots were kept frozen at −70 °C until the end of the study, when they were analysed. Measurements of cytokines, C-X-C motif chemokines (CXCL), and adhesion molecules (IL-1β, IL-4, IL-6, IL-8, IL-10, IL-17, IFN-γ, TNF-α, sICAM1, sVCAM1, sE-Selectin, CXCL1, CXCL9, CXCL10, and CXCL12) were performed with Quantikine ELISA kits (R&D Systems, Inc., Minneapolis, MN, USA), according to the manufacturer’s instructions. sP-Selectin was measured with the human sP-Selectin/CD62P immunoassay kit from R&D Systems. Details regarding the sensitivity, specificity, linearity, and intra-/inter-assay precision are available in the manufacturer’s website (http://www.rndsystems.com). White blood cell counts, hemoglobin F, reticulocytes, biochemical markers of kidney and liver function, and C-reactive protein were measured in freshly collected blood samples.

### Statistical analysis

The statistical analysis was performed with GraphPad Prism software version 6 (GraphPad Software Inc., San Diego, CA, USA). The Wilcoxon matched-pairs signed rank test was used whenever two experimental groups were compared. When indicated, the one-sample Wilcoxon signed rank test was used to compare the median of a group to a specified value. Nonparametric analysis of data from paired groups obtained in three time points was performed by the Friedman test, followed by the Dunn’s post test. *P* values <0.05 were considered significant. Medians and confidence intervals (CI) are presented.

## Results

### Effect of MTX treatment on pain intensity and frequency of acute VOC episodes

**Fig. 1:**
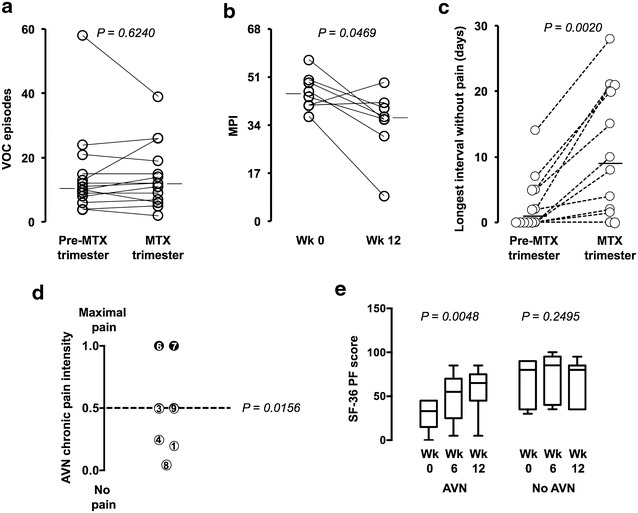
Methotrexate effect on VOC frequency, pain, and physical functioning. **a** Comparison of the number of VOC episodes recorded during a trimester that preceded the study and during the 12-week MTX treatment. **b** McGill Pain Index (MPI): the scores obtained at the end of the 12-week course of MTX were compared to those from week 0. The results of the McGill pain questionnaire administered to patients that reported at the last follow-up visit general clinical improvement (graded ≥5 in a scale of 0–10) are shown. **c** Longest uninterrupted pain-free period in between crises as perceived by the patients. **d** Chronic pain intensity: *Each circle* represents a patient, whose number is indicated. The *position of the circles* indicates the intensity of the avascular osteonecrosis-associated pain reported by individual patients after 12 weeks of MTX treatment plotted on a scale, in which 0 represents no pain and 1.0 represents the maximal baseline chronic pain. The 50% cutoff is indicated by the *dashed line* and data points at or under this level are shown as *open circles*. The *P* value was calculated with the Wilcoxon signed-rank test by comparing the median intensity of 0.5 (95% CI 0.05–1.0) at the last follow-up visit to the maximal baseline chronic pain before treatment (1.0). **e** SF-36 physical functioning (PF) subscale: scores obtained at weeks 0, 6, and 12 for patients with and without avascular osteonecrosis (AVN) are plotted in 25–75% interquartile boxes with whiskers set at 5 and 95 percentiles. The medians are indicated

The number of VOC episodes during the 12-week methotrexate treatment was not significantly different from that recorded in a preceding trimester: the medians were 12 (95% CI 6–26) and 10.5 (95% CI 6–21), respectively, (*P* = 0.6240; Fig. 1a). However, patients were asked to rate the general effectiveness of MTX treatment in a scale of 0–10 at the last follow-up visit of the study: 8 out 14 individuals reported general improvement in their condition, with a score ≥5 (median 7, 95% CI 5–8.5, n = 8). Their effectiveness perception was supported by the lower score obtained with the McGill Pain Questionnaire at week 12, compared to the one administered at week 0. The median McGill Pain Index (MPI) dropped 18.9% at 12 weeks in these 8 patients, from 45 (95% CI 37–57) to 36.5 (95% CI 9–49) (*P* = 0.0469, n = 8, Fig. 1b). The Additional file 1: Table S1 shows the MPI results for all 14 participants by the end of the study. The median MPI decreased 27.2% from 49.5 (95% CI 37–57) to 36 (95% CI 9–45) in 8 patients, and increased 22.5% from 40 (95% CI 37–52) to 49 (95% CI 48–57) in 4 cases. This index was nearly unchanged (<3%) in 2 individuals. Overall, there was no significant difference (*P* = 0.931).

In addition to acute painful VOC episodes, patients may also experience nociceptive chronic pain caused by avascular osteonecrosis that is difficult to quench, is resistant to opioids and hardly ceases in-between crises [27]. Seven of the 14 patients who entered the study had avascular osteonecrosis of the femoral and/or humeral heads demonstrated by magnetic resonance imaging (Table 1). Five of them (#1, #3, #4, #8 and #9) referred a chronic pain reduction ≥50% after MTX treatment (*P* = 0.015). Only 2 patients (#6 and #7) did not perceive any benefit at week 12 (Fig. 1d).

The osteonecrosis pain data was combined with the MPI results, in order to compile a list of responders and nonresponders to MTX therapy, which revealed pain relief in 71% of the patients (10/14). This response was heterogeneous, probably reflecting the overlapping occurrence of acute and chronic pain (Table 2). Three patients (#1, #3 and #8) exhibited the highest relief because they had both lower MPI and decrease of osteonecrosis pain. Patient #1 evolved from having continuous hip pain, even between VOC episodes, to a situation in which there were periods without any pain.

**Table 2:** Methotrexate therapy effect on pain intensity according to the McGill pain index and avascular osteonecrosis chronic pain evaluation

Patient no.	McGill pain index reduction^a^	Avascular osteonecrosis pain reduction^b^	Composite effect^c^
Responder			
14	Yes (11.8%)	N/A	Yes
10	Yes (18.2%)	N/A	Yes
11	Yes (20.0%)	N/A	Yes
12	Yes (36.8%)	N/A	Yes
13	Yes (31.4%)	N/A	Yes
8	Yes (75.7%)	Yes (≥50%)	Yes
1	Yes (24.5%)	Yes (≥50%)	Yes
3	Yes (34.8%)	Yes (≥50%)	Yes
4	No (−19.5%)	Yes (≥50%)	Yes
9	No (−2.4%)	Yes (≥50%)	Yes
Nonresponder			
6	No (−25.6%)	No	No
7	No (−9.6%)	No	No
2	No (−29.7%)	N/A	No
5	No (−2.6%)	N/A	No

*N/A* not applicable

^a^Negative values indicate increase of the index

^b^Avascular osteonecrosis pain as defined in Fig. 1d

^c^Composite effect means either avascular osteonecrosis pain reduction or lower McGill pain index or both

Patient #8 had bilateral osteonecrosis of the femoral and humeral heads, with previous hip joint replacement on the right side. His clinical picture improvement during MTX therapy allowed him to walk without crutches.

It is interesting that there were two patients (#4 and #9) who did not experience pain reduction associated to acute VOC episodes, but had ≥50% decrease in chronic osteonecrosis pain. Patient #4 illustrates well this category of response: at the end of the study, her MPI was 19% higher. However, the chronic pain completely disappeared in her right shoulder and reduced 50% in her hips. She also regained mobility of the gleno-humeral joint that had been greatly compromised by the humeral head necrosis. Patient #9 had a similar MPI before and after MTX treatment, but had 50% reduction of the osteonecrosis pain intensity in his left hip joint.

To all 10 patients that were classified in Table 2 as MTX responders and two of those that fell into the MTX nonresponder category (#6 and #7), it was asked to define the longest uninterrupted period without pain at home. As indicated in Fig. 1c, the majority (10 individuals) responded that they achieved longer pain-free periods under MTX treatment, as compared to the trimester that preceded the study [median: 9 days (95% CI 1.5–21) vs 1 day (95% CI 0–5.0), *P* = 0.0020, n = 12]. Six of the participants said that they felt continuous persistent pain all the time before the MTX treatment, four of whom (#1, #3, #13 and #14) admitted gaining pain-free days under MTX therapy.

### Quality of life measurement after methotrexate treatment

There was a transient drop in the scores of the SF-36 role emotional subscale at week 6 of the MTX treatment, but patients scored similarly when results from week 0 and 12 were compared (Additional file 1: Figure S1A–E). Among the 8 domain subscales of the questionnaire [23], only the one that measures physical functioning was significantly and persistently improved by the methotrexate treatment. In concordance with the pain reduction reported by most patients, a 44.4% median gain of physical function could be identified in the entire study group at week 12 (Additional file 1: Figure S1A). This finding was also evident in the MTX responder subgroup as defined in Table 2, which had a median score 105.8% higher by the end of the study (Additional file 1: Figure S1B). Patients with avascular osteonecrosis scored almost double of the initial level of physical functioning by the end of the MTX treatment [Median: 65 (95% CI 5–85) vs 33 (95% CI 0–45), *P* = 0.004, n = 7], while those without avascular osteonecrosis scored similarly at weeks 0 and 12 (*P* = 0.249, n = 7) (Fig. 1e).

There was no major change pattern related to depression in the studied patients as assessed by the Beck Inventory [25] (Additional file 1: Figure S2). Four individuals in the avascular osteonecrosis subgroup kept their depression status (#1, #7, #8 and #9), one moved from moderate to mild (#3), one moved from mild to moderate (#4), and one changed from minimal to mild (#6). The results were similar for the group without avascular osteonecrosis, with unaltered status in three individuals (#12, #13 and #14), improvement from mild to minimal depression in one case (#11), and change from minimal to mild in two occasions (#2 and #5) and from minimal to moderate in another (#10).

### Methotrexate therapy effect on inflammatory markers

Table 3 summarizes the cytokine, adhesion molecule and chemokine plasma levels at weeks 0, 6 and 12 of the study. C−Reactive Protein (CRP) was also used as an additional unspecific inflammation marker in 9 out of the 14 patients, being moderately to highly increased in the blood of most individuals tested at week 0, except one (#9). There was a median drop in CRP concentration of 58.4% in six individuals (#7, #8, #11, #12, #13 and #14) and an increase in two patients (#6 and #10: 90.5 and 44%, respectively). The CRP levels remained close to normal baseline in one case (#9). The median CRP levels ranged from 0.84 mg/dL (week 0) to 0.55 mg/dL (week 12) (*P* = 0.359, Table 3).

**Table 3:**
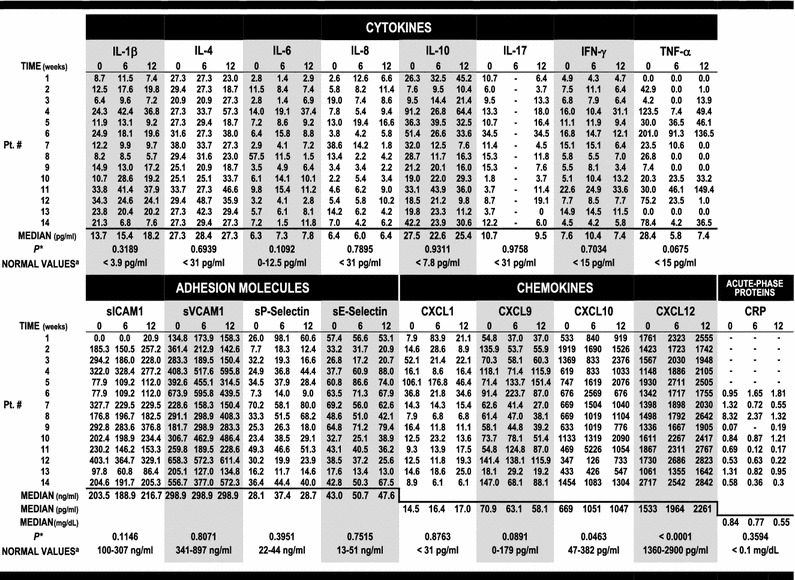
Methotrexate effect on cytokines, adhesion molecules, chemokines and CRP

* The *P* value was calculated by the Friedman test for all markers considering data obtained in three time points (weeks 0, 6 and 12), except in the cases of IL-17 and the inflammatory marker CRP, for which the measurements from the first and last time points were compared by the Wilcoxon matched-pairs signed rank test

^a^Normal plasma values for IL-1β, IL-4, IL-6, IL-8, IL-10, IL-17, IFN-γ, TNF-α, sICAM1, sVCAM1, sP-Selectin, sE-Selectin, CXCL1, CXCL9, CXCL10, and CXCL12 were obtained from 10-54 healthy donors for each ELISA kit by the manufacturer (http://www.rndsystems.com). CRP blood concentration was measured by high-sensitivity nephelometry, and normal reference values were described in the National Health and Nutrition Examination Survey (NHANES) [28]

**Fig. 2:**
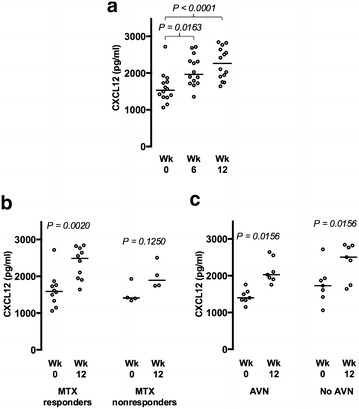
Methotrexate-induced CXCL12 up-regulation. **a** CXCL12 plasma levels steadily increase during MTX therapy in all patients. **b** CXCL12 measurements in MTX responder and MTX nonresponder individuals as defined in Table 2. **c** CXCL12 levels in patients with and without avascular osteonecrosis. Data collected in weeks 0, 6, and 12 of the MTX treatment are presented in **a** and from weeks 0 and 12 in **b** and **c**. The medians are indicated

Among the inflammatory markers tested, MTX treatment had a highly significant impact on two chemokines, that possess both chemotactic and angio-modulatory properties. The most significant effect was exerted on the pro-angiogenic factor CXCL12, with a plasma concentration upward trend registered in all patients (*P* < 0.0001) (Table 3; Fig. 2a). The increase of CXCL12 levels occurred significantly in MTX-responder patients (*P* = 0.002) and trended upward in nonresponders (*P* = 0.125), irrespectively of the presence of avascular osteonecrosis (*P* = 0.015; Fig. 2b, c). The chemokine CXCL10 concentration followed a similar pattern described above, with an upward trend induced by MTX treatment (*P* = 0.0463) (Table 3; Additional file 1: Figure S4B–D).

High plasma levels of IL-1β and IL-10 were recorded among all patients at the 3 study point determinations. There was no significant up- or down-regulation in plasma concentration of these two and twelve additional inflammatory proteins measured at weeks 0, 6, and 12 of the MTX treatment, including major cytokines, chemokines, and adhesion molecules: IL-1β, IL-4, IL-6, IL-8, IL-10, IL-17, IFN-γ, TNF-α, sICAM1, sVCAM1, sP-Selectin, sE-Selectin, CXCL1, and CXCL9. These results are illustrated in Table 3 alongside normal reference values. In the case of TNF-α, methotrexate induced a down-regulation of the cytokine plasma levels in 60% of the patients at week 12 of the study (8 out of 12), with a more pronounced decrease in the middle time point, from 28.4 pg/ml at week 0 to 5.8 pg/ml at week 6 (*P* = 0.0753) (Table 3; Additional file 1: Figure S4A).

Finally, there was no significant alteration in hematological and biochemical parameters tested during the study (Additional file 1: Figure S3).

### MTX adverse events

Considering that all patients were in routine use of hydroxyurea, plus concomitant use of other drugs, such as opioids, antibiotics, chlorpromazine or carbamazepine, it was difficult to identify/separate any adverse effect related to MTX itself. According to the National Cancer Institute (NCI) Common Terminology Criteria for Adverse Events v3.0 (CTCAE) [29], three patients developed low blood platelet counts: grade 1 (Patient #5: 91,000/μl) and grade 4 (Patient #10: 24,000/μl and #13: 11,000/μl) attributable to hydroxyurea that returned to normal levels in a few days after discontinuation of that medication. All these three patients had previous history of hydroxyurea-induced hematological toxicity. Nevertheless, MTX treatment was interrupted for 1–2 weeks and resumed thereafter. Patient #2 had an episode of seizure (grade 2), possibly related to SCD. The following infectious events were observed: dengue fever (Patient #8), common cold (Patient #12), pharyngitis (Patient #11 and #3), sinusitis (Patient #12), pneumonia (Patient #8), and FUO (Patient #6). Patients received antibiotics when indicated and MTX administration was temporarily suspended. Patient #2 had a central venous line infection (grade 5). Although catheter and blood cultures became negative after antibiotic treatment, the infection reappeared later on, and the patient died of pneumonia and septic shock 11 days after the end of the study. In this last case there was a drop in WBC counts (grade 2: 2070/μl), platelets (grade 3: 28,000/μl), and lymphocyte counts (grade 3: 270/μl), being difficult to differentiate between peripheral sepsis consumption or additional myelosuppression.

## Discussion

Ten out of fourteen patients exhibited some degree of response to methotrexate, ranging from mild pain improvement (Patient #14) to dramatic outcomes (Patient #8, who had a 75% MPI drop and over 90% reduction of his osteonecrosis pain). The best responders were often among those with osteonecrosis (Patients #1, #3, #4, #8, and #9). Furthermore, pain relief was accompanied by functional gain, most notably in those who had osteonecrosis. It is worth mentioning that the SF-36 physical functioning subscale used in this study has also been applied as a stand-alone assessment tool in similar conditions, that may involve physical limitation and pain, including peripheral arterial disease and hip fracture [30, 31]. The disappearance or substantial reduction of osteonecrosis-associated pain in five out of seven patients was remarkable, and occurred even when vaso-occlusion crises have not subsided. Three of the five individuals with osteonecrosis who responded to methotrexate (Patients #1, #3, and #4) reported persistent pain before the MTX treatment, with virtually no pain-free intervals. A therapeutic alternative for this complication is highly desirable, given that its low responsiveness to opioids leaves little to offer to the patients other than surgical replacement of the affected bone area [6, 27, 32].

The use of MTX was associated to the patient’s perception of occurrence of longer uninterrupted pain-free periods in between crises. It is not surprising that any pain-free period produced by MTX use would be seen as a tremendous gain, notably for those that previously had continuous chronic pain.

Although not evaluated in this study, the MTX beneficial effect is likely to be independent of a mechanism of reduction of sickling episodes similar to that induced by hydroxyurea [33, 34]. Methotrexate did not have, in this study, any significant effect on hemoglobin F levels and reticulocyte counts.

Rheumatoid arthritis and sickle cell disease are similar in that they both have an inflammatory component [4, 11–13, 15]. Rheumatoid arthritis is an autoimmune disorder associated with significant morbidity, caused by immune-mediated destruction of synovial surfaces and progressive joint damage [15]. The use of low-dose aminopterin as a tissue reactive suppressor was attempted for the first time close to 75 years ago in seven arthritis patients, with symptom improvement reported in 6 individuals [35]. At the time, the immune modulatory effect of aminopterin was not known and its effectiveness on pain control despite the lack of analgesic activity was puzzling. Aminopterin was soon replaced by the closely related molecule methotrexate that became the mainstay of the rheumatoid arthritis treatment [36]. The potentially beneficial effect of methotrexate in sickle cell disease can be inferred from a few clinical reports in which it was used to treat co-existing juvenile rheumatoid arthritis [37, 38]. MTX is an antifolate that acts by inhibiting folate-dependent enzymes, one of the most affected being AICAR (5-aminoimidazole-4-carboxamide ribonucleotide) transformylase, resulting in accumulation of AICAR [39]. AICAR inhibits AMP deaminase directly and the AICAR dephosphorylated form inhibits adenosine deaminase, leading to net tissue accumulation of AMP and/or adenosine [36].

Extracellular AMP is converted into adenosine by the ecto-5′-nucleotidase CD73 expressed on the endothelial surface [40]. Adenosine signals through G-coupled receptors (A_1_R, A_2A_R, A_2B_R, and A3) and is a powerful immune modulator to different cells known to participate of the inflammatory response in rheumatoid arthritis and sickle cell disease, such as M1 macrophages and neutrophils [4, 13, 41]. Adenosine down-regulates pro-inflammatory cytokine production in macrophages and decreases ROS generation and phagocytic activity in neutrophils, which also lose their selectin- and integrin-mediated adhesion to the endothelium [41]. It is noteworthy that adenosine immune modulation plays a protective role in several animal models of ischemia/reperfusion injury involving different target organs [9, 42]. A similar injury mechanism has been suggested to occur in sickle cell disease [4]. One could envisage that MTX-induced adenosine and its AMP precursor would leak into the local microcirculation from multiple cell types and counteract the pro-inflammatory effect of the products released during hemolysis.

A decrease in plasma levels of TNF-α, the master inflammatory regulator, was observed in weeks 6 and 12 of the MTX treatment, with a more pronounced down-regulation in the middle of the study but it did not reach significance in either time point (*P* = 0.075 for week 6 and *P* = 0.899 for week 12; Additional file 1: Figure S4A). Conversely, up-regulation of two chemokines, CXCL10 and CXCL12, was detected. Although CXCL10 is expected to promote inflammation by attracting CXCR3-positive immune cells to active sites [43], it was not possible to find an augmentation of inflammatory effector molecules after MTX treatment. A possible explanation is that methotrexate-induced extracellular adenosine blunts CXCL10 chemotactic activity through A_2a_ receptor activation and heterologous desensitization of CXCR3 [44–46].

The up-regulation of CXCL12 may be a central point to the understanding of the MTX clinical impact on SCD patients. CXCL12 is a powerful angiogenic factor that recruits endothelial progenitor cells from the bone marrow and have regenerative and tissue protective effects in ischemic conditions, such as myocardial infarction [47, 48]. Importantly, it counteracts and overrides angiostatic stimuli, such as those from the CXCL10/CXCR3 axis [49]. CXCL12 also attracts mesenchymal stem cells to the inflammation sites, where they differentiate into osteoblasts and chondrocytes halting inflammatory bone destruction [50]. CXCL12 may decrease in situ inflammation through these highly immunosuppressive mesenchymal stem cells or by direct induction of Th1 repolarization [51, 52]. Thus, CXCL12 could account for the clinical improvement produced by MTX by mobilizing pro-angiogenic bone marrow cells, thereby limiting the local damage of ischemic episodes and their associated pain. Any consequent reduction of inflammatory bone destruction and perhaps even occurrence of bone regeneration could be particularly beneficial to patients with avascular osteonecrosis.

It is not known how MTX activates the above-mentioned chemokines in SCD, nor if it is a direct or indirect effect. Nevertheless, MTX has shown promise in reducing the often opioid-resistant osteonecrosis-associated pain in this present series. Our findings suggest CXCL12 as a putative marker that could mediate a possible MTX-induced limitation of ischemia–reperfusion damage in sickle cell disease.

The long-term implications of the MTX treatment in sickle cell disease are unknown. There is mounting evidence that deregulated angiogenesis may be an important component of a complex pathophysiology, affecting the course of complications as varied as leg ulcers, proliferative retinopathy, pulmonary hypertension and moyamoya syndrome [53–58]. The CXCL12 up-regulation described in this report will likely have a context-dependent impact on the evolution of the disease. Mobilization of bone marrow cells might have positive implications if they limit inflammation [51, 52] or if they promote neovascularization of ischemic areas, such as leg ulcers [58]. However, mobilized fibrocytes might contribute to interstitial lung disease and pulmonary hypertension [53, 55], and a pro-angiogenic environment might accelerate the development of certain complications, such as proliferative retinopathy [57]. However, we need to consider that treatment with hydroxyurea has also anti-angiogenic activity, most likely mediated by HIF-1α down-regulation [56, 57], which might not be helpful to wound healing and revascularization of infarcted areas. Yet, hydroxyurea is the only drug currently approved by the FDA for the treatment of SCD because of its overall benefit to the patients [33]. Similarly, it remains to be determined if the putative therapeutic value of methotrexate in sickle cell disease overweighs any possible negative effect.

Finally, activation of the A_1_ receptor was shown to reduce central nociceptive signaling in the spinal cord and it is possible that the MTX-induced systemic release of adenosine may decrease the neuropathic pain component in SCD patients [59–61].

## Conclusions

Methotrexate did not change significantly the number and intensity of acute pain episodes in SCD patients but reduced chronic pain from avascular osteonecrosis, with perception of longer pain-free periods, and quality of life improvement. This is a pilot study that should be interpreted cautiously because of the small number of patients included and the fact that it has not being designed in a blinded, randomized format. It remains to be determined if a better clinical outcome could be achieved by recruiting younger and less compromised patients, prolonging the treatment period, and optimizing dosing and administration route. These are all relevant points that can only be meaningfully addressed in a larger prospective clinical trial. Methotrexate has been around for almost 80 years [18, 35], and its successful use beyond oncology indicates that it is an old dog that can learn new tricks!

## Additional file

**Additional file 1.** Additional results: Impact of methotrexate on hematological, kidney, and liver function parameters. **Figure S1.** Effect of methotrexate treatment on quality of life. **Figure S2.** Impact of methotrexate treatment on depression status. **Figure S3.** Impact of methotrexate treatment on hematological and biochemical parameters. **Figure S4.** Effect of methotrexate on TNF-α and CXCL-10. **Table S1.** McGill Pain Index.

## Abbreviations

SCD: sickle cell disease
MTX: methotrexate
VOC: vaso-occlusive pain crises
QOL: quality of life
AVN: avascular osteonecrosis
ROS: reactive oxygen species
TLR4: toll-like receptor 4
WPBs: Weibel–Palade bodies
NSAID: nonsteroidal anti-inflammatory drugs
CI: confidence intervals
MPI: McGill pain index
CRP: C−reactive protein
NCI CTCAE: National Cancer Institute Common Terminology Criteria for Adverse Events
AICAR: 5-aminoimidazole-4-carboxamide ribonucleotide
NHANES: National Health and Nutrition Examination Survey
PF: physical functioning
RP: role physical
BP: bodily pain
GH: general health
MH: mental health
RE: role emotional
SF: social functioning
V: vitality
RBC: red blood cell
RET: reticulocyte
HBF: hemoglobin F
HGB: hemoglobin
WBC: white blood cells
LYM: lymphocytes
NEU: neutrophils
PLT: platelets
GOT: serum glutamic-oxaloacetic transaminase
GPT: serum glutamate-pyruvate transaminase
BUN: blood urea nitrogen
CRN: creatinine
CXCL: C-X-C motif chemokines
