# Supplementary material for: Low-dose methotrexate in sickle-cell disease: a pilot study with rationale borrowed from rheumatoid arthritis
Source: Exp Hematol Oncol. 2017 Jun 19;6:18. doi: 10.1186/s40164-017-0078-1 (PMC5474854; doi:10.1186/s40164-017-0078-1)
Supplement: Supplementary file 1 — Additional file 1. Additional results: Impact of methotrexate on hematological, kidney, and liver function parameters. Figure S1. Effect of methotrexate treatment on quality of life. Figure S2. Impact of methotrexate treatment on depression status. Figure S3. Impact of methotrexate treatment on hematological and biochemical parameters. Figure S4. Effect of methotrexate on TNF-α and CXCL-10. Table S1. McGill Pain Index. [file 40164_2017_78_MOESM1_ESM.pdf]

Low-dose methotrexate in sickle-cell disease: a pilot study with rationale borrowed from rheumatoid arthritis

Brandalise *et al.*

Additional files

Supplementary results: Impact of methotrexate on hematological, kidney, and liver function parameters.

Supplementary figures:

Figure S1. Effect of methotrexate treatment on quality of life.

Figure S2. Impact of methotrexate treatment on depression status.

Figure S3. Impact of methotrexate treatment on hematological and biochemical parameters.

Figure S4. Effect of methotrexate on TNF-α and CXCL-10.

Supplementary table:

Table S1. McGill Pain Index.

SUPPLEMENTARY RESULTS

IMPACT OF METHOTREXATE ON HEMATOLOGICAL, KIDNEY, AND LIVER FUNCTION PARAMETERS

Supplementary Figure S3, panel A, shows that there was not a significant impact on common hematological parameters, such as: total red and white blood cell counts and measurements of total hemoglobin, reticulocytes, lymphocytes, and neutrophils. There was an increase in median hemoglobin F levels [From 8.65% (95% CI: 2.8-22) at week 0 to 11% (95% CI: 4.7-27) at week 12] and in median platelet counts [From 262.5 x 10^3/μl (95% CI: 165-367) at week 0 to 328 x 10^3/μl (95% CI: 209-506) at week 12] but the upward trends were not significant (Friedman test P = 0.1378 and 0.1092, respectively). In addition, the stability of the blood concentration of glutamic-oxaloacetic and glutamate-pyruvate transaminases, urea and creatinine during the 12 week MTX treatment revealed an apparent lack of hepatic and kidney toxicity (Suppl. Figure S3).

SUPPLEMENTARY FIGURES

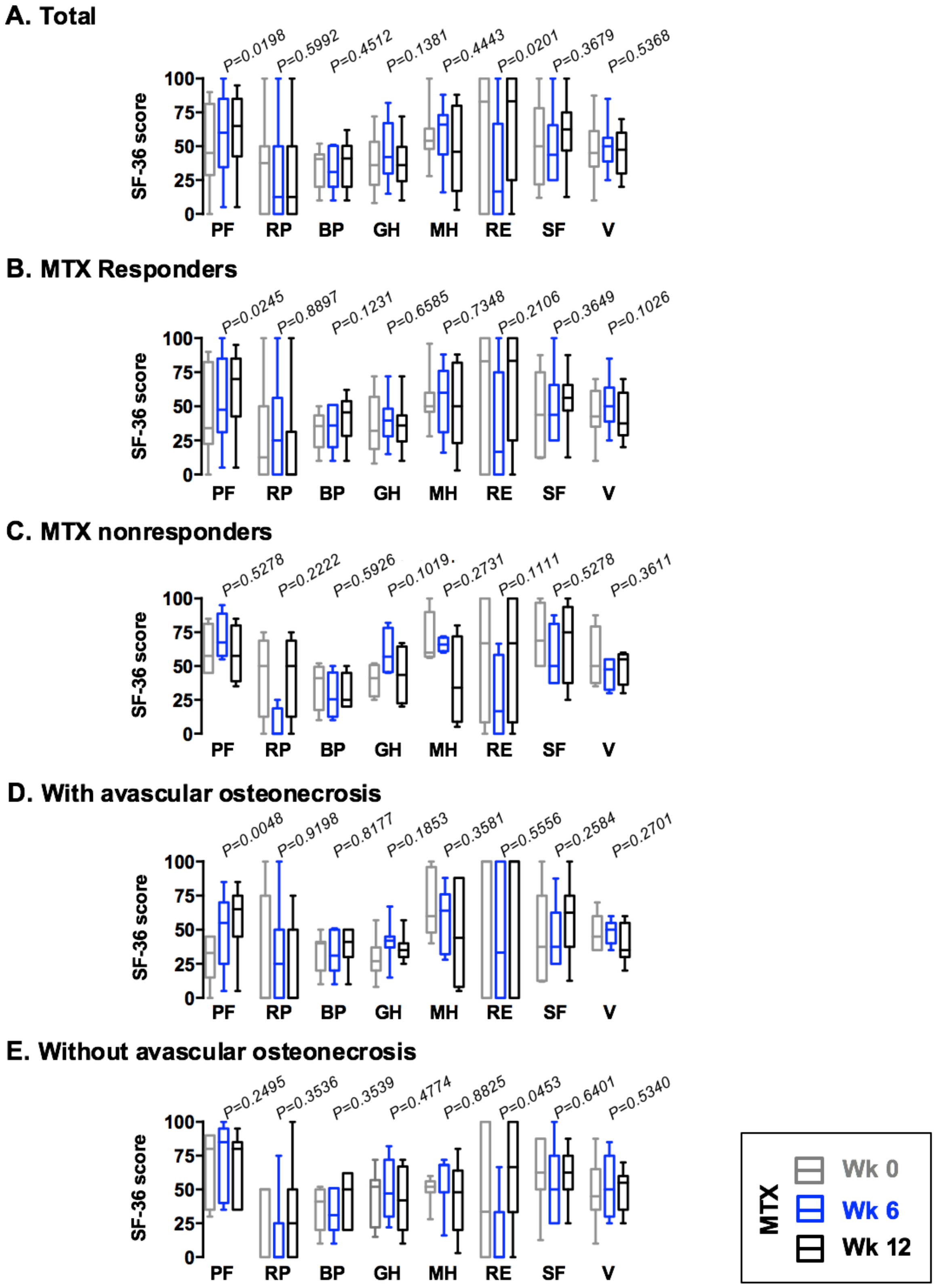

FIGURE S1. Effect of methotrexate treatment on quality of life.

The Brazilian Portuguese translation of the SF-36 questionnaire was administered to patients at week 0 (gray line boxes), week 6 (blue line boxes) and week 12 (black line boxes). Score results are presented in 25-75% interquartile boxes, with the median indicated, and whiskers set at 5-95 percentiles for: all 14 patients (panel A), MTX responders (panel B), MTX nonresponders (panel C), patients with avascular osteonecrosis (panel D), and without avascular osteonecrosis (panel E). SF-36 domains are indicated as follows: physical functioning (PF), role physical (RP), bodily pain (BP), general health (GH), mental health (MH), role emotional (RE), social functioning (SF), and vitality (V). The Friedman ANOVA *P* value is shown for each domain in all panels.

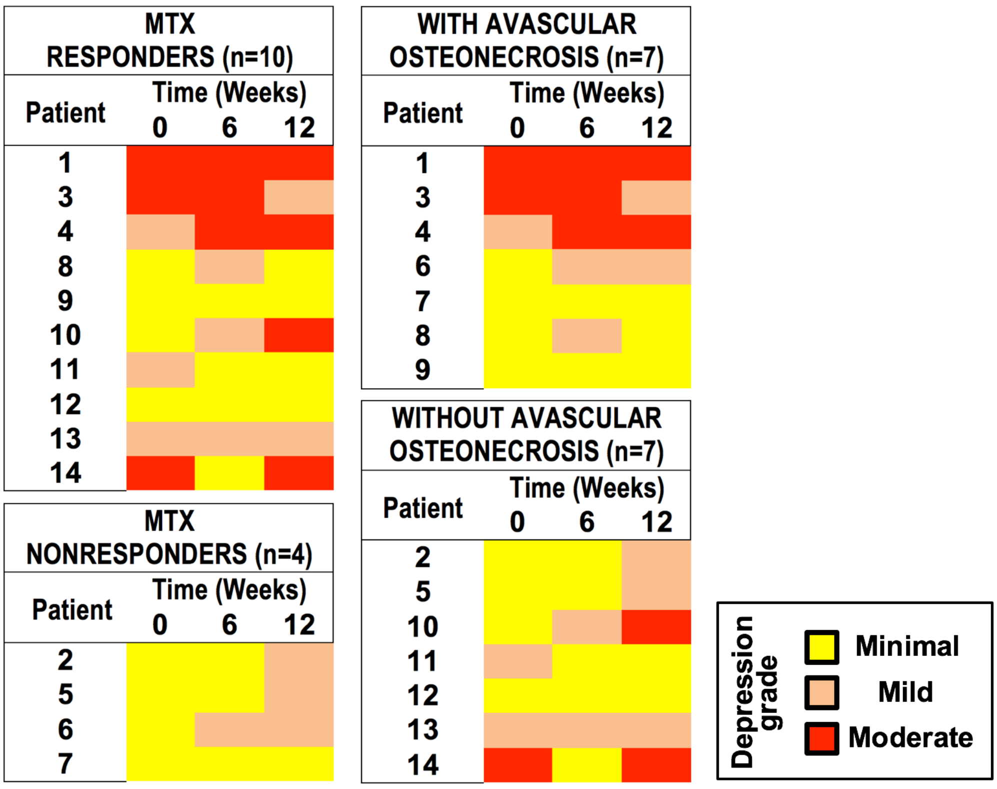

FIGURE S2. Impact of methotrexate treatment on depression status.

The Brazilian Portuguese translation of the Beck Depression Inventory was applied to patients at weeks 0, 6, and 12 of the study as indicated. The depression status is shown in a 3-level heat map for responders and nonresponders to the MTX treatment, and in patients with and without avascular osteonecrosis.

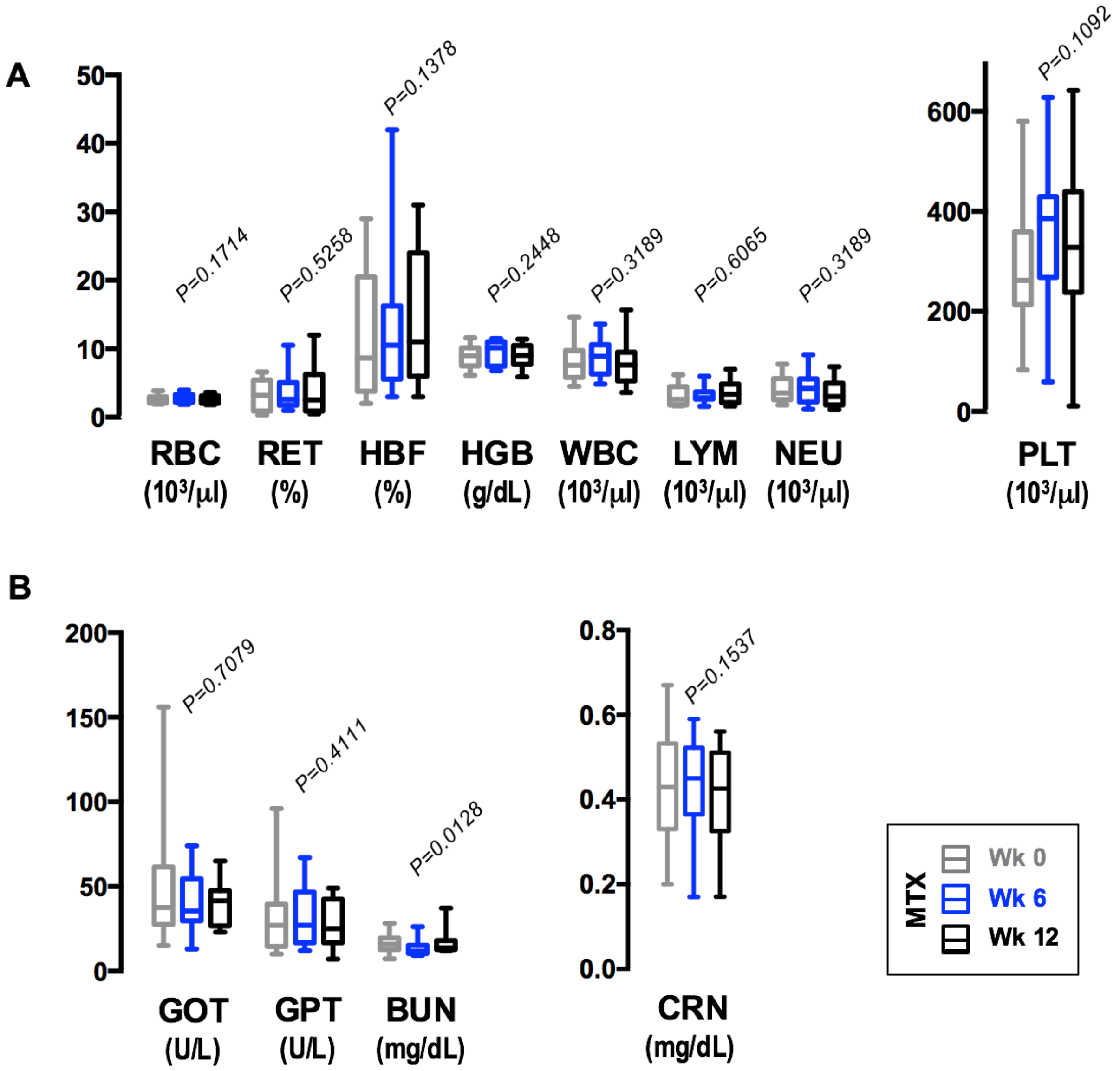

FIGURE S3. Impact of methotrexate treatment on hematological and biochemical parameters.

The figure shows the comparative analysis of hematological **(A)** and biochemical **(B)** parameters measured at three time points of the MTX treatment: week 0 (gray line boxes), week 6 (blue line boxes), and week 12 (black line boxes). Results are plotted in 25-75% interquartile boxes, with the median indicated, and whiskers set at 5-95 percentiles. The Friedman ANOVA *P* value for each parameter is also indicated.

RBC: Red Blood Cell; RET: Reticulocyte; HBF: Hemoglobin F; HGB: Hemoglobin; WBC: White Blood Cells; LYM: Lymphocytes; NEU: Neutrophils; PLT: Platelets; GOT: Serum Glutamic-Oxaloacetic Transaminase; GPT: Serum Glutamate-Pyruvate Transaminase; BUN: Blood urea nitrogen; CRN: Creatinine.

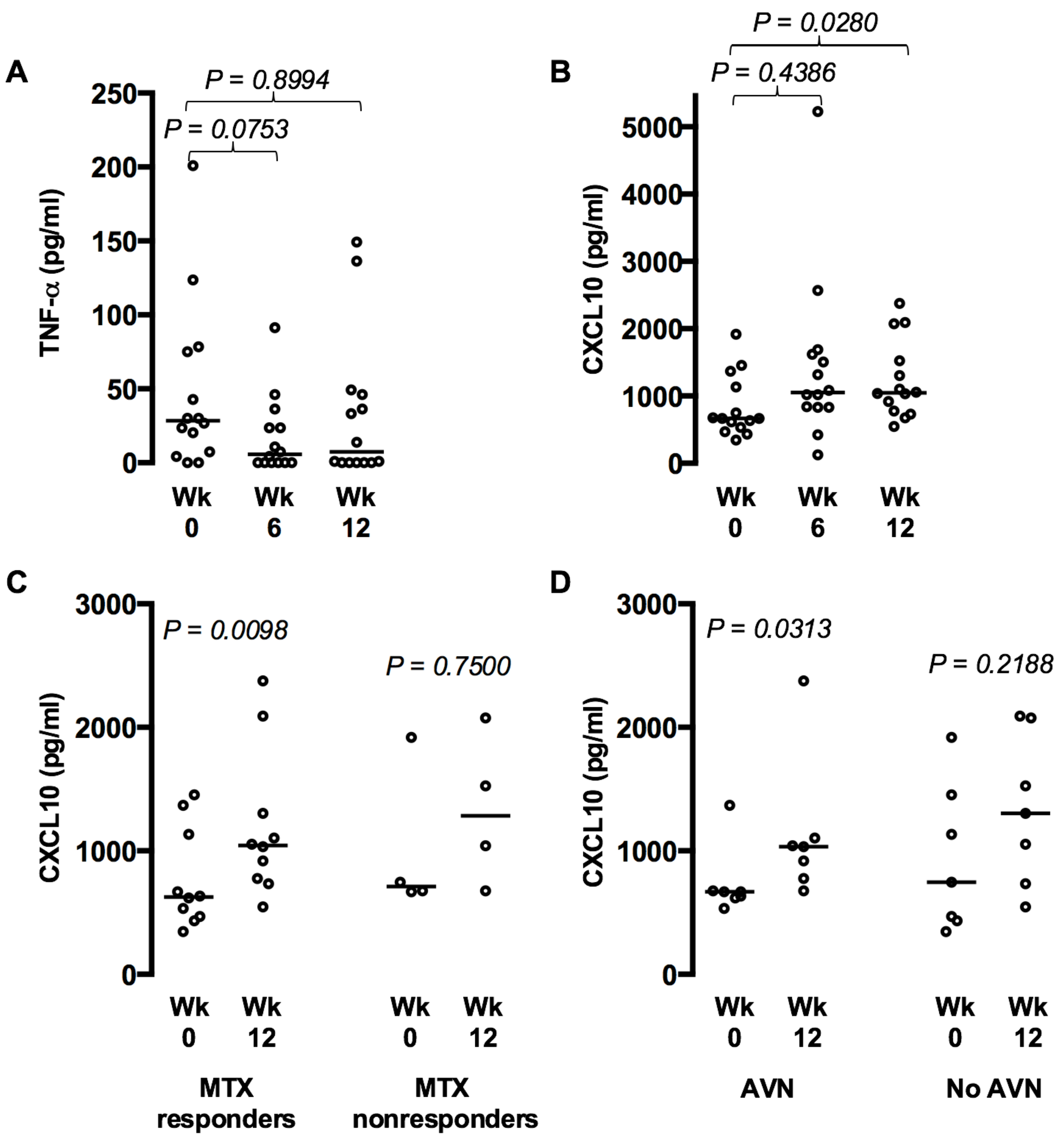

FIGURE S4. Effect of methotrexate on TNF-α and CXCL-10.

A. The TNF-α plasma concentrations of all 14 patients are presented for measurements made at weeks 0, 6 and 12 of the MTX therapy. **B.** The CXCL10 plasma levels of all 14 patients are shown for the same three time points used for TNF-α analysis. **C.** The comparison of CXCL10 median plasma levels in weeks 0 and 12 of the study is presented in MTX responder patients (n=10) and MTX nonresponder individuals (n=4). **D.** The CXCL10 median plasma concentration in patients with (n=7) and without (n=7) avascular osteonecrosis is shown for weeks 0 and 12 of the MTX treatment. The *P* values were calculated by using Friedman ANOVA followed by Dunn's post test in panels A and B and by the Wilcoxon matched-pairs signed rank test in panels C and D. Medians are indicated in all panels.

SUPPLEMENTARY TABLE

TABLE S1. McGill Pain Index.

	TIME (weeks) →	0	6	12
PATIENT #	1	49	34	37
	2	37	46	48
	3	46	28	30
	4	41	46	49
	5	38	43	39
	6	39	59	49
	7	52	53	57
	8	37	15	9
	9	41	45	42
	10	44	42	36
	11	50	38	40
	12	57	48	36
	13	51	56	35
	14	51	39	45
MEDIAN MPI →		45	44	39.5
	*P**		0.9311	

* The *P* value was calculated by the Friedman test considering data obtained in three time points (Weeks 0, 6 and 12).
